# How to select patients and timing for rectal indomethacin to prevent post-ERCP pancreatitis: a systematic review and meta-analysis

**DOI:** 10.1186/s12876-017-0599-4

**Published:** 2017-03-15

**Authors:** Jianhua Wan, Yuping Ren, Zhenhua Zhu, Liang Xia, Nonghua Lu

**Affiliations:** Department of Gastroenterology, The First Affiliated Hospital of Nanchang University, 17 Yongwaizheng Street, Nanchang, Jiangxi 330006 People’s Republic of China

**Keywords:** Indomethacin, Pancreatitis, ERCP, Meta-analysis

## Abstract

**Background:**

Acute pancreatitis is a severe complication of endoscopic retrograde cholangiopancreatography (ERCP). Previous meta-analyses have shown that indomethacin effectively prevents this complication; however, the data are limited. We performed a systematic review and meta-analysis to clarify the applications for rectal indomethacin.

**Methods:**

A systematic search was performed in June 2016. Human prospective, randomized, placebo-controlled trials that compared rectally administered indomethacin with a placebo for the prevention of post-ERCP pancreatitis (PEP) were included. A meta-analysis was performed using a random-effects model to assess the outcomes (PEP) using Review Manager 5.0.

**Results:**

Seven randomized controlled trials met the inclusion criteria (*n* = 3013). The overall incidence of PEP was significantly lower after prophylactic administration of rectal indomethacin than after administration of the placebo (RR, 0.58, 95% CI, 0.40–0.83; *P* = 0.004). A subgroup analysis was performed for rectal indomethacin administration compared to a placebo in high-risk patients (RR, 0.46; 95% CI, 0.32–0.65; P < 0.00001) and average-risk patients (RR, 0.75; 95% CI, 0.46–1.22; P = 0.25) and for administration before ERCP (RR, 0.56; 95% CI, 0.39–0.79; *P* = 0.001) and after the procedure (RR, 0.61; 95% CI, 0.26–1.44; *P* = 0.26).

**Conclusions:**

This meta-analysis indicated that prophylactic rectal indomethacin is not suitable for all patients undergoing ERCP but it is safe and effective to prevent PEP in high-risk patients. In addition, rectal indomethacin administration before ERCP is superior to its administration after ERCP for the prevention of PEP.

**Electronic supplementary material:**

The online version of this article (doi:10.1186/s12876-017-0599-4) contains supplementary material, which is available to authorized users.

## Background

Acute pancreatitis is the most common complication after post-endoscopic retrograde cholangiopancreatography (ERCP). The incidence rate of post-ERCP pancreatitis (PEP) ranges between 1.6 and 15.7%, with an overall average of 3.5% [1]. While most cases of PEP are mild, 10-20% of patients may develop severe acute pancreatitis with adverse outcomes and many intractable complications. Therefore, the social and financial burdens caused by PEP need to be addressed. One study noted that the possible pathogenesis of PEP includes both increased pressure and radiocontrast exposure, which contribute to injury in the PEP model [2]. However, the detailed pathogenesis of PEP remains unknown, and no specific or effective treatment for pancreatitis has been developed. Numerous factors have been found to be correlated with the development of PEP, including patient-related factors, such as an age of less than 50 years, female sex, pancreas divisum, sphincter of Oddi dysfunction (SOD), a history of recurrent pancreatitis (≥2 episodes) or history of PEP, and procedure-related factors, such as pancreatic sphincterotomy, precut sphincterotomy, difficult cannulation, pancreatic duct injection or endoscopist experience [3].

Over the past two decades, many methods have been used to prevent PEP, including pharmacologic prevention and mechanical-related interventions. Treatment has been unsatisfactory with the exception of the use of rectal nonsteroidal anti-inflammatory drugs (NSAIDs) and prophylactic pancreatic stents. A network meta-analysis based on existing randomized controlled trials (RCTs) showed that rectal NSAIDs are one of the most efficacious agents for preventing PEP [4]. However, prophylactic stent placement is not cost-effective in patients at average risk for the development of PEP [5]. NSAIDs, especially indomethacin, are potent inhibitors of phospholipase A2 activity, which can regulate proinflammatory mediators such as prostaglandins, leukotrienes and platelet-activating factors in the initial inflammatory cascade of acute pancreatitis [6]. Therefore, prophylactic rectal indomethacin administration to prevent PEP is biologically plausible. A meta-analysis by Rustagi et al.[7] showed that only the rectal route resulted in a significant benefit for the prevention of PEP compared to non-rectal administration of indomethacin. Compared to other methods, rectal indomethacin is less expensive and easily administered, leading to potential beneficial effects in PEP.

Recent clinical trials and a large number of meta-analyses have suggested the promising outcomes of indomethacin use. The European Society of Gastrointestinal Endoscopy and the Japanese Society of Hepato-Biliary-Pancreatic Surgery guidelines recommended routine rectal administration of indomethacin in unselected (both high-risk and average-risk) patients to prevent PEP [8, 9]. However, recent high quality RCTs have revealed that prophylactic rectal indomethacin did not reduce the incidence or severity of PEP in consecutive patients undergoing ERCP [10]. It is necessary to reconsider the selection of suitable patients for prophylactic rectal indomethacin after ERCP. A survey from 29 countries reported using NSAIDs for PEP prophylaxis was not widely accepted by endoscopists performing ERCP due to the lack of convincing scientific evidence [11]. Therefore, a larger sample meta-analysis should examine the benefits of rectal indomethacin for PEP.

## Methods

### Literature search

We followed standard criteria for performing and reporting a meta-analysis of RCT studies [12]. A systematic search of PubMed, EMBASE and the Cochrane library (including CENTRAL) was performed to identify potentially relevant publications (through June 2016). Keywords included indomethacin, pancreatitis and ERCP. The search was restricted to human studies, and no language restrictions were set. In addition, the reference lists of all retrieved articles, as well as reviews and abstracts from recent conferences, were manually searched. When the same or similar patient studies were included in several publications, only the most recent or informative report was selected for analysis.

### Study selection

Studies were initially selected based on their titles and abstracts. Two reviewers (JH.W. and YP.R.) independently screened all abstracts to determine whether the studies met the inclusion criteria. Differences were resolved by a third investigator (L.X.). Studies were considered eligible if they met the following criteria: (1) studies that examined the efficacy and safety of prophylactic rectal indomethacin use for PEP; (2) studies that were prospective and randomized; (3) studies in humans; and (4) data that were not duplicated in another article. Studies were excluded if (1) the study design was retrospective or the study was not an RCT or (2) unadjusted estimates were reported.

### Data abstraction and quality assessment

To ensure homogeneity in data gathering and entry, the data extraction was conducted by two experienced investigators working independently (JH.W. and YP.R.). A third investigator (L.X.) was called upon to resolve any differences so that complete consensus was reached for all of the main variables to be assessed in the analysis. Data were recorded as follows: the first author’s last name (year of publication), country, setting, study design, size of the trial, outcomes, intervention, inclusion criteria, exclusion criteria, definition of PEP, complications and study quality (recorded in Table 1). The quality of the included studies was assessed independently by two reviewers (JH.W. and YP.R.) using the Cochrane Collaboration tool for assessing the risk of bias [13] (Additional file 1 Figure S1). The grading system contains the following criteria: random sequence generation, allocation concealment, blinding of participants and personnel, blinding of outcome assessment, incomplete outcome data, selective reporting and other bias. Each trial was given an overall summary assessment of low, unclear, or high-risk of bias.

**Table 1:** Characteristics of Studies Included in Meta-analysis

Study	Year	Location	Indomethacin (n)	Placebo (n)	Number of PEPs (n)	Intervention	Definition of PEP
Montaño Loza *et al.*	2007	Mexico	75	75	16	100 mg indomethacin 2 h before ERCP	Pain, Amylase > 3 times
Sotoudehmane sh *et al.*	2007	Iran-Single centre	245	245	22	100 mg indomethacin immediately before ERCP	Pain, Amylase > 3 times admission
Elmunzer *et al.*	2012	US-Multicentre	295	307	79	two 50-mg indomethacin after ERCP	Pain, Amylase > 3 times admission > 2 nigh
döbrönte *et al.*	2014	Hungary-Multicentre	347	318	42	100 mg indomethacin 10–15 min before ERCP	Pain, Amylase > 3 times a prolognation of admission, CT/MRI
Andrade-Dávila *et al.*	2015	Mexico	82	84	21	100 mg indomenthacin after ERCP	Pain, Amylase > 3 times admission > 2 nigh
Patai *et al.*	2015	USA-Single centre	270	296	55	100 mg indomethacin within 1 before ERCP	Pain, Amylase > 3 times
Levenick *et al.*	2016	USA-Single centre	223	226	27	two 50-mg indomethacin during ERCP	Pain, Amylase > 3 times admission > 2 nigh

### Statistical analysis

Statistical analysis of the Relative Risk (RR) with the 95% confidence interval (CI) was used as a common measure of the association between rectal indomethacin and PEP across studies. Taking both within-study and between-study variabilities into account, we used a random-effects model, which is more conservative than a fixed-effects model, to aggregate data and obtain the overall effect size and 95% CI. Heterogeneity across studies was assessed by performing *X* ^2^ tests (assessing the P value) and calculating I^2^, which is a quantitative measure of inconsistency across studies. Studies with an I^2^ of 25 to 50% were considered to have low heterogeneity; studies with an I^2^ of 50to 75% were considered to have moderate heterogeneity; and studies with an I^2^ > 75% were considered to have high heterogeneity. If I^2^ > 50%, potential sources of heterogeneity were identified by sensitivity analyses conducted by omitting one study at a time and investigating the influence of a single study on the overall pooled estimate. Publication bias was examined by Egger’s test and Begg’s test. All calculations were conducted with Review Manager V 5.0 software (provided by the Cochrane Collaboration, Oxford, UK) and Stata version 12.0 (Stata Corporation, College Station, TX, USA). All P values were two-sided, and the significance level was 0.05.

## Results

### Identification of eligible studies

**Fig. 1:**
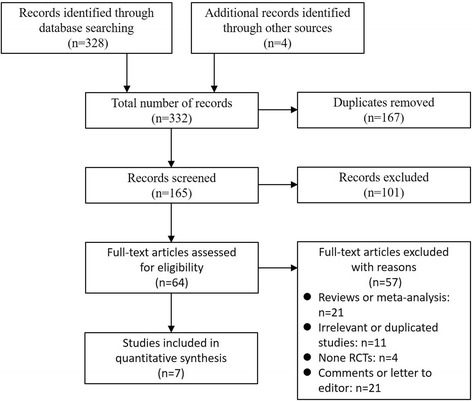
Identification of eligible studies from different databases

Based on our search criteria, we identified 332 papers from MEDLINE/PubMed, EMBASE and the Cochrane Central Register of Controlled Trials in the Cochrane Library. Of these articles, 167 duplicate articles were removed. Of those articles, the majority were excluded after reviewing titles and abstracts, mainly because they were reviews, letters, comments, retrospective studies or not relevant to our analysis, leaving 64 papers that appeared to meet our selection criteria. Of those papers, 21 were excluded because they were reviews or meta-analyses; 11 were excluded for irrelevance or were duplicate studies; 4 studies were excluded because they were non-RCTs and 21 papers were excluded because they were comments or letters to the editor. Finally, a total of 7 RCTs with 3013 participants were included in the meta-analysis [10, 14–19]. A detailed flowchart of the selection process is shown in Fig. 1.

### Study characteristics

The main characteristics of the studies in the meta-analysis are presented in Table 1. These studies were published between 2007 and 2016. Among the 7 studies, 3 were conducted in America [10, 15, 16], 2 studies were conducted in Mexico [14, 19] and the remaining 2 studies were conducted in Hungary [18] and Iran [17]. All studies were published in English language journals. All studies used a total dose of 100 mg of rectal indomethacin, but included pre-ERCP and post-ERCP administration. Two studies selected patients with an elevated baseline risk of PEP.

### Main results

**Fig. 2:**
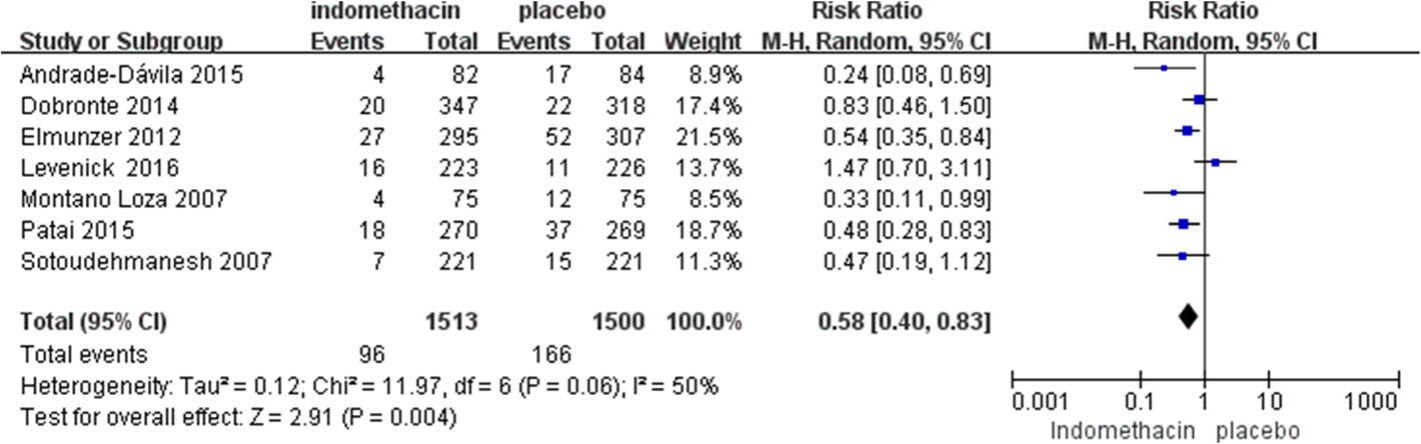
Forest plot of the overall rate of PEP treatment with rectal indomethacin

As the primary outcome, the incidence of PEP was measured in all 7 studies. The RR was evaluated between rectal indomethacin and a placebo for the prevention of PEP. The Mantel-Haenszel pooled RR for PEP after prophylactic administration of rectal indomethacin compared to the placebo was 0.58 (95% CI, 0.40–0.83; *P* = 0.004; Fig. 2), corresponding to an absolute risk reduction of 4.8 percentage points (number needed to treat [NNT] to prevent one episode of post-ERCP pancreatitis was 21) and a relative risk reduction of 43%. Statistically, heterogeneity was observed (I^2^ = 50%; *P* = 0.06), which may have occurred due to the following three factors: two studies selected patients with an elevated baseline risk of PEP, two studies used the prophylactic placement of pancreatic stents for most of the patients, and the timing of administration of rectal indomethacin differed among the studies. Therefore, the random effects model was used.

### Subgroup analyses

**Fig. 3:**
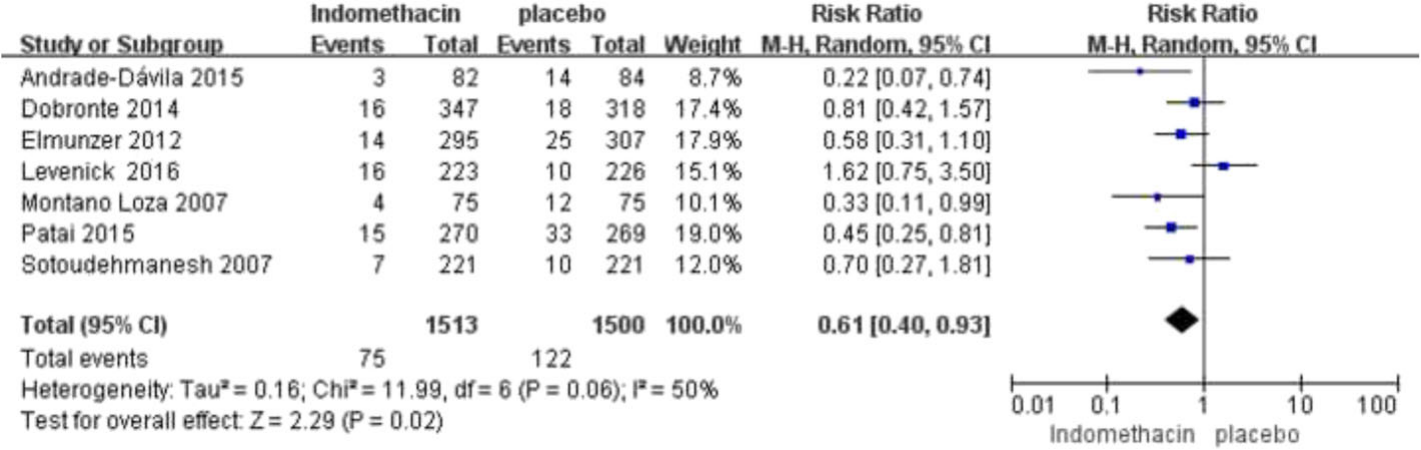
Forest plot of mild vs. moderate-to-severe PEP treated with rectal indomethacin

**Fig. 4:**
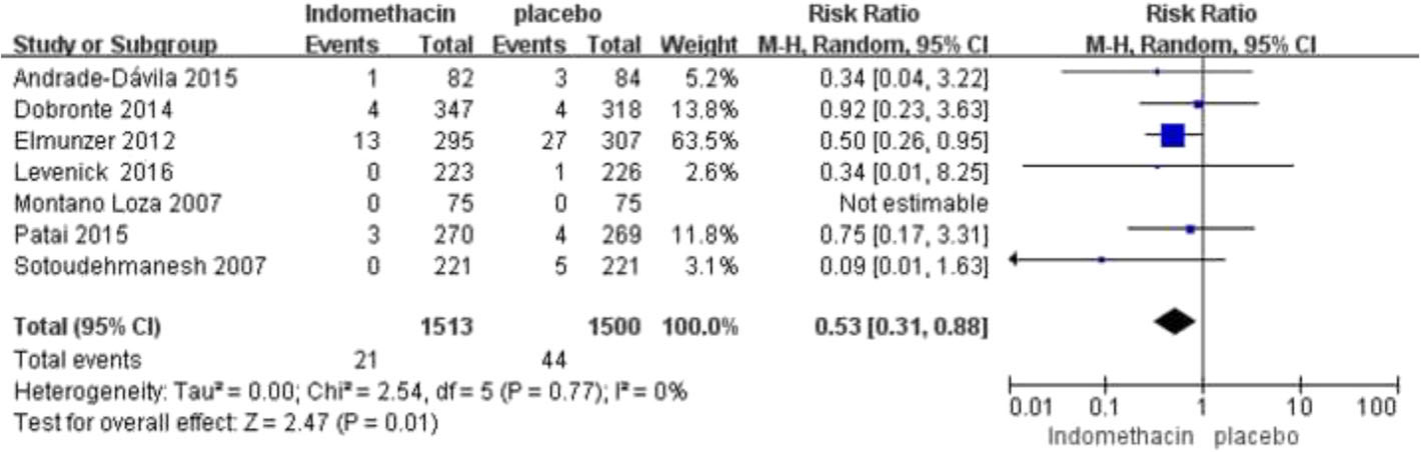
Forest plot of mild vs. moderate-to-severe PEP treated with rectal indomethacin

In our subgroup analysis by severity of PEP, 7 studies (*n* = 3013; weight, 74.5%) using rectal indomethacin for the prevention of mild PEP showed a significant difference (RR, 0.61; 95% CI, 0.40–0.93; *P* = 0.02), and similarly, 6 studies (*n* = 3013; weight, 25.5%) using rectal indomethacin for prevention of moderate-to-severe PEP showed a significant benefit (RR, 0.53; 95% CI, 0.31–0.88; *P* = 0.01) (Fig. 3 and 4). In summary, rectal indomethacin can reduce the incidence of mild and moderate-to-severe PEP.

**Fig. 5:**
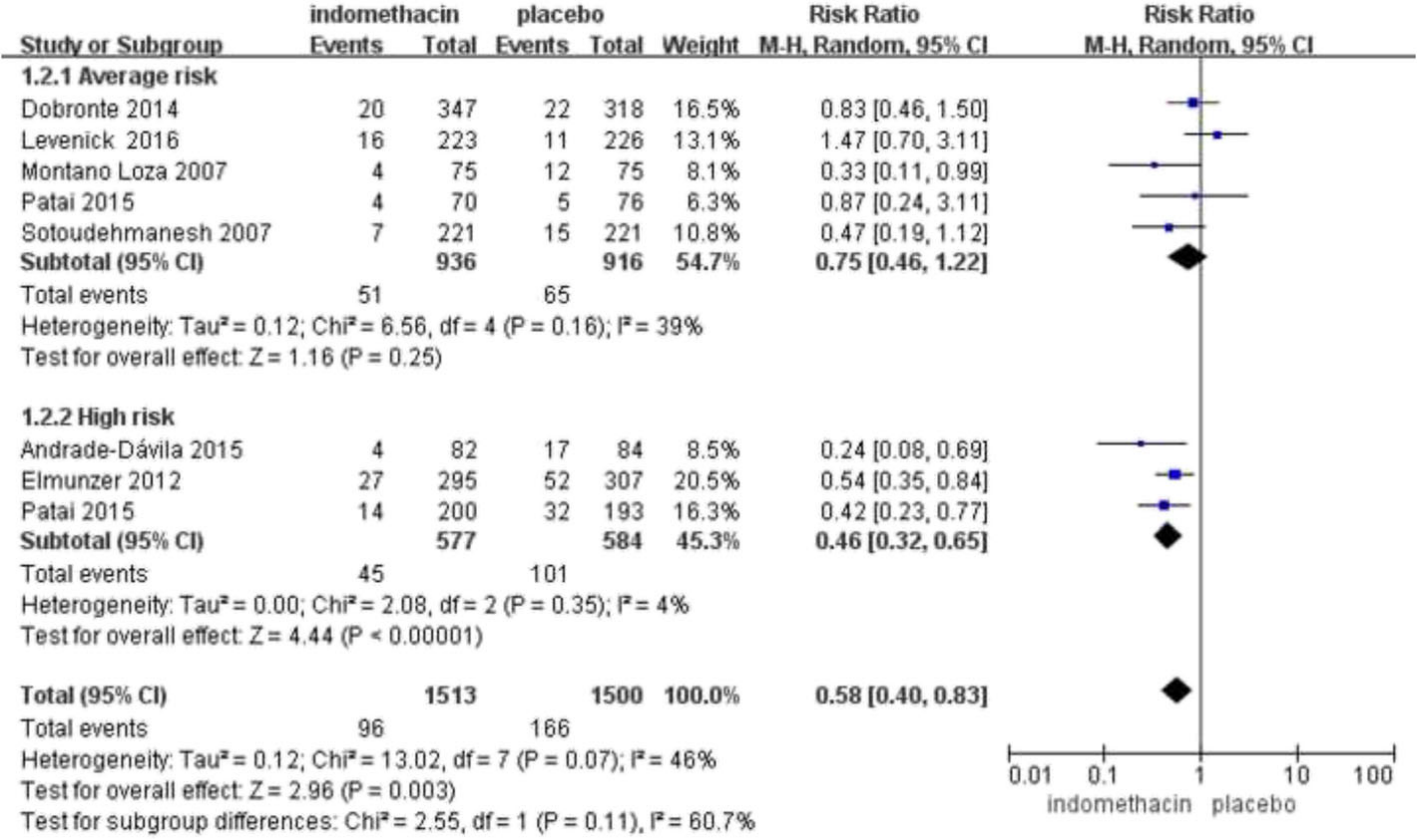
Forest plot of patients at average risk vs. high risk for PEP treated with rectal indomethacin

When comparing patients at average risk and high risk for PEP, rectal indomethacin showed a significant overall reduction in the incidence of PEP only in the high-risk patients (3 studies with 1161 patients; weight, 45.3%) (RR, 0.46; 95% CI, 0.32–0.65; *P* < 0.00001), whereas pooled data from 5 studies (*n* = 1852; weight, 54.7%) involving patients at average risk for PEP showed that rectal indomethacin had no significant benefit (RR, 0.75; 95% CI, 0.46–1.22; *P* = 0.25) (Fig. 5). The NNT to prevent 1 episode of PEP in the high-risk patient group was 10.

**Fig. 6:**
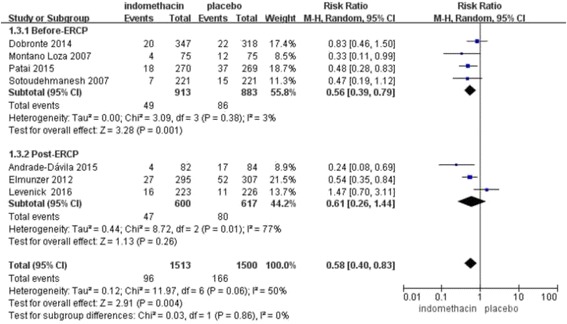
Forest plot of the timing of administration of rectal indomethacin for PEP

In the subgroup analysis of the timing of administration of rectal indomethacin, pooled data from 4 studies (*n* = 1796; weight, 55.8%) in which rectal indomethacin was administered before ERCP showed a statistically significant difference in the occurrence of PEP related to this timing (RR, 0.56; 95% CI, 0.39–0.79; *P* = 0.001). However, 3 studies (*n* =1217; weight, 44.2%) in which rectal indomethacin was administered after the procedure showed no significant benefit (RR, 0.61; 95% CI, 0.26–1.44; *P* = 0.26) for PEP prophylaxis (Fig. 6).

**Fig. 7:**
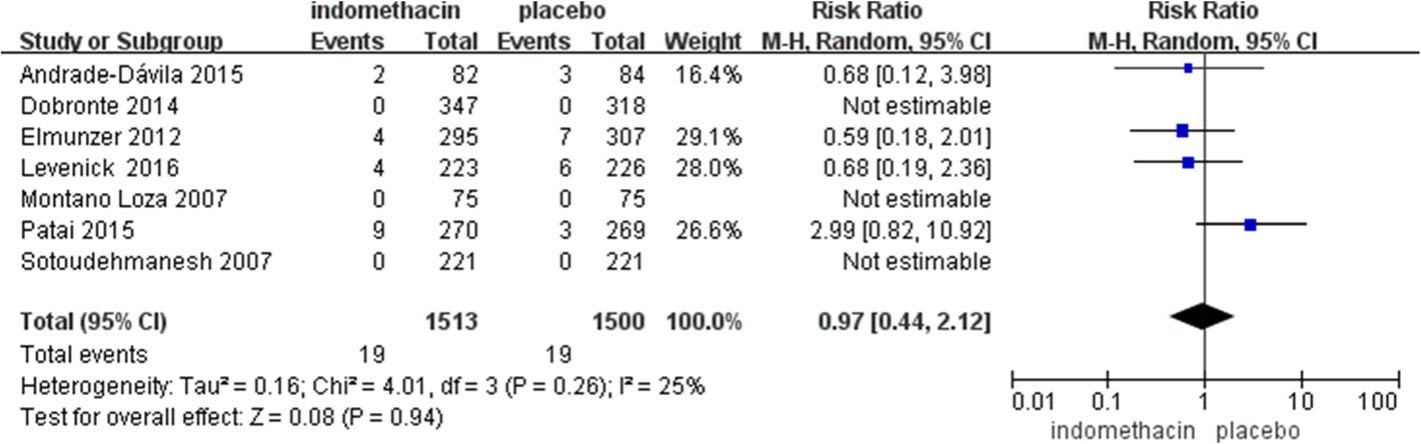
Forest plot of bleeding as an adverse clinical event in the treatment of PEP using rectal indomethacin

Only four studies reported bleeding as an adverse event that was potentially related to indomethacin. The results showed no statistical significance between the two groups (RR, 0.97; 95% CI, 0.44–2.12; *P* = 0.94) (Fig. 7).

### Sensitivity Analysis

When a single study involved in the meta-analysis was deleted each time, the results of meta-analysis remained unchanged, indicating that the results of the present meta-analysis were stable.

### Publication bias

**Fig. 8:**
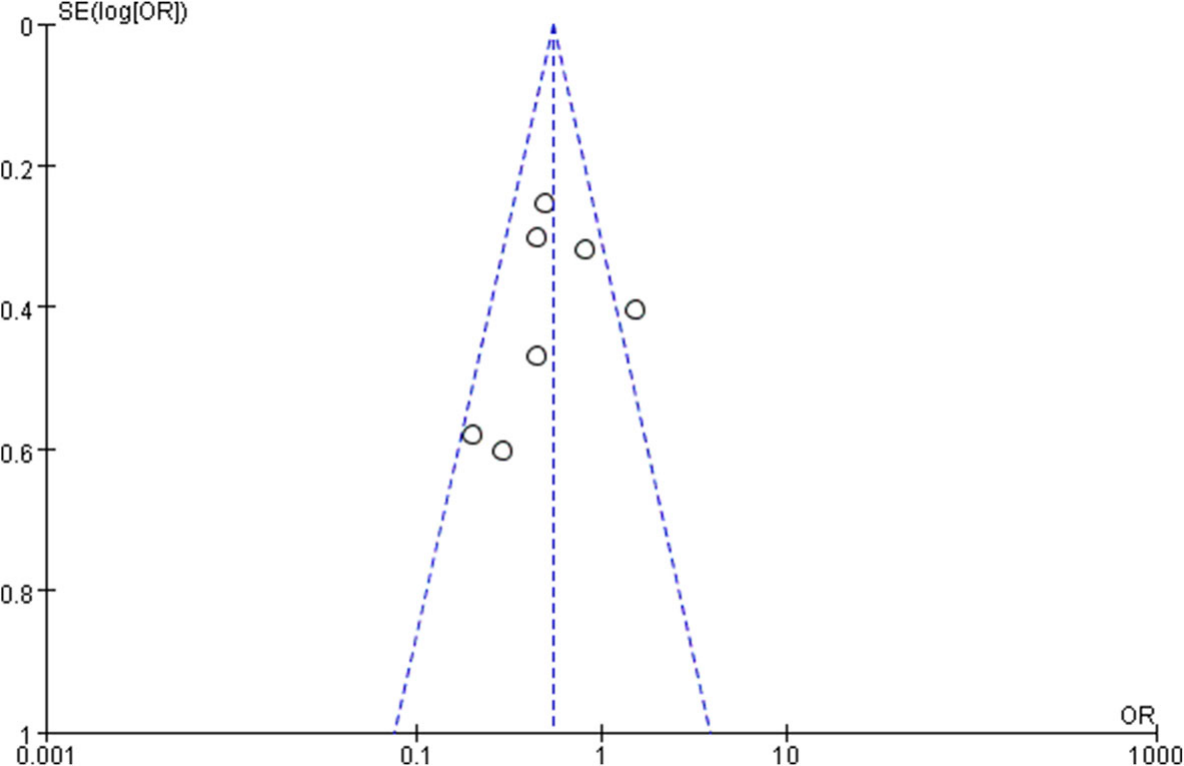
Funnel Plot to measure publication bias of the meta-analysis

A funnel plot showed that the studies were reasonably well scattered (Fig. 8). There was no statistical evidence of publication bias among studies by using both Egger’s regression asymmetry test (*P* = 0.61) (Additional file 2 Figure S2) and the Begg’s adjusted rank correlation (*P* = 0.37) (Additional file 3 Figure S3).

## Discussion

In this meta-analysis, we found that rectal indomethacin is generally more effective than a placebo for preventing PEP in patients undergoing ERCP. It reduces the incidence of PEP by nearly 43%, with an NNT of approximately 22 subjects. However, some studies included patients of different classification, leading to the presence of clinical heterogeneity. Therefore, this result is not very persuasive. Previous meta-analyses all concluded that rectal indomethacin was superior to a placebo for preventing PEP in both average- and high-risk patients undergoing ERCP [7, 20–28]. However, those meta-analyses included only a small number of patients who used indomethacin, which reduces the precision of the comparative results, and their conclusions were limited. Three of those meta-analyses included only 3 or 4 studies [20, 21, 23]. Another meta-analysis included indomethacin and other NSAIDs, such as diclofenac, or other routes of administration [7, 22, 24–28]. Compared to the results of previous meta-analyses, the results of the present meta-analysis included more recent RCTs that were different from the RCTs included in the previous analyses. In our subgroup analysis of average- and high-risk patients, rectal indomethacin was not effective in patients at average risk for PEP. Recently, an RCT from a single center showed that prophylactic rectal indomethacin did not reduce the incidence or severity of PEP in consecutive patients undergoing ERCP [10]. In this study, patients were deliberately not categorized into high- and low-risk groups for PEP. Hence, rectal indomethacin should be applied as the choice for patients at high risk for PEP, considering its effectiveness, economy and side effects. Similarly, Elmunzer et al. [15] showed that two 50-mg doses of rectal indomethacin significantly reduced the risk of PEP from 16.9% in those receiving the placebo to 9.2% in those receiving indomethacin for patients at high risk for PEP, including 82.3% of patients who had a clinical suspicion of SOD dysfunction. It should be noted that in this study, the authors placed a pancreatic stent in 246 patients in the indomethacin group (83.4%) and in 250 individuals in the placebo group (81.4%).

In our subgroup analysis of post-ERCP and pre-ERCP prophylactic administration, rectal indomethacin was not effective in patients when administered post-ERCP. Previous research has found that the peak plasma concentration of indomethacin is reached 30 min after rectal administration, when bioavailability is complete [29]. The elimination half-life of indomethacin is 4.5 h. When the drug was used before ERCP, the peak level was achieved at the desirable time. Theoretically, therefore, rectal indomethacin may be more effective before the ERCP than after the procedure. A meta-analysis by Rustagi et al. [7] in 2014 found that NSAID administration before ERCP had a greater benefit than administration after the procedure. Recently, Luo et al. found that the strategy of prophylactic pre-ERCP administration of rectal indomethacin for all patients was superior to the strategy of purposeful rectal indomethacin after ERCP in only high-risk patients for reducing the risk of PEP [30]. Therefore, the timing of administration of rectal indomethacin should be before rather than after ERCP.

Of note, no differences in adverse events potentially attributable to rectal indomethacin treatment were observed, suggesting that indomethacin is a safe pharmacologic agent for the prevention of PEP. Four studies reported bleeding as an adverse event, but statistical significance was not achieved. Three patients died from severe PEP in 3 studies, all of which occurred in the placebo group. Other adverse events also occurred in these 3 studies.

The present meta-analysis has some limitations. First, low-quality and small number of studies were included. Second, this meta-analysis exhibits statistical homogeneity. Andrade-Dávila et al. [19] and Elmunzer et al. [15] enrolled only patients at high risk for PEP, whereas the other 5 studies enrolled average-risk patients. Third, studies differed in their definition of PEP and did not always adhere to the Cotton criteria. Lastly, this meta-analysis did not consider the influence of prophylactic pancreatic stents. Patients in 2 studies underwent prophylactic placement of pancreatic stents [10, 15].

In summary, this is the first meta-analysis to suggest that rectal indomethacin is not suitable for all patients undergoing ERCP and should be recommended for preventing PEP in high-risk patients before ERCP. In addition, larger multi-center RCTs are still needed to determine the role of rectal indomethacin in low-risk patients.

## Conclusion

Although this meta-analysis indicates that prophylactic rectal indomethacin is not suitable for all patients undergoing ERCP, it is safe and effective for the prevention of PEP in high-risk patients. In addition, administration of rectal indomethacin before ERCP is superior to administration after ERCP for the prevention of PEP. In conclusion, it is necessary to recommend rectal indomethacin before ERCP for the prevention of PEP in high-risk patients.

## Additional files

Additional file 1: Figure S1. Risk of bias summary in this review. Green: Low risk, Yellow: Unclear, Red: High risk. (PNG 7 kb)
Additional file 2: Figure S2. Egger’s publication bias plot. (TIF 998 kb)
Additional file 3: Figure S3. Begg’s funnel plot of RCTs. (TIF 998 kb)

## Abbreviations

CI: Confidence interval
ERCP: Endoscopic retrograde cholangiopancreatography
NNT: Number needed to treat
NSAIDs: Nonsteroidal anti-inflammatory drugs
PEP: Post-ERCP pancreatitis
RCTs: Randomized controlled trials
RR: Relative Risk
SOD: Sphincter of Oddi dysfunction
