# Supplementary figures and images for: How to select patients and timing for rectal indomethacin to prevent post-ERCP pancreatitis: a systematic review and meta-analysis
Source: BMC Gastroenterol. 2017 Mar 15;17:43. doi: 10.1186/s12876-017-0599-4 (PMC5353805; doi:10.1186/s12876-017-0599-4)

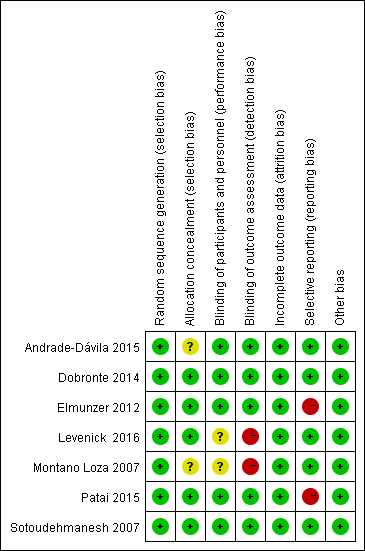

Supplement: Additional file 1: Figure S1. — Risk of bias summary in this review. Green: Low risk, Yellow: Unclear, Red: High risk. (PNG 7 kb) [file 12876_2017_599_MOESM1_ESM.png]

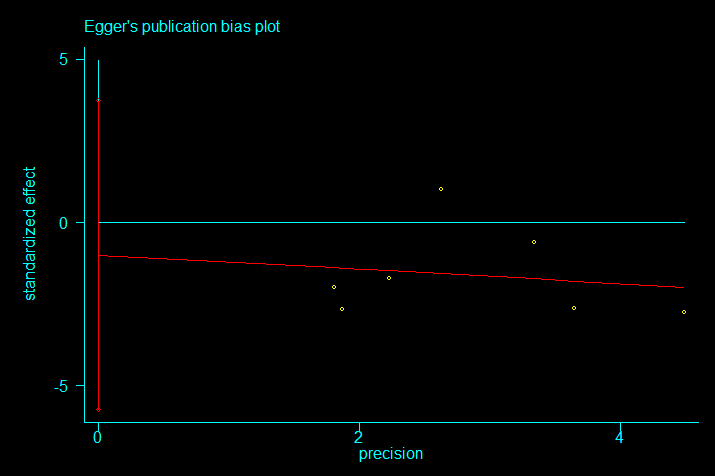

Supplement: Additional file 2: Figure S2. — Egger’s publication bias plot. (TIF 998 kb) [file 12876_2017_599_MOESM2_ESM.tif]

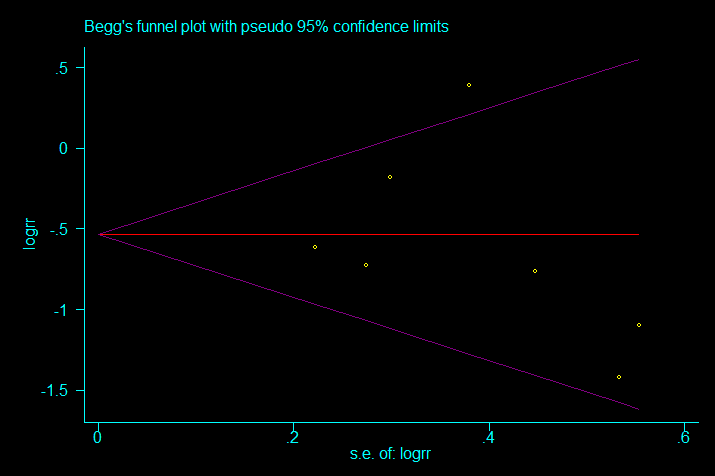

Supplement: Additional file 3: Figure S3. — Begg’s funnel plot of RCTs. (TIF 998 kb) [file 12876_2017_599_MOESM3_ESM.tif]
